# Genetic and Epigenetic Etiology of Inflammatory Bowel Disease: An Update

**DOI:** 10.3390/genes13122388

**Published:** 2022-12-16

**Authors:** Sara Jarmakiewicz-Czaja, Magdalena Zielińska, Aneta Sokal, Rafał Filip

**Affiliations:** 1 Institute of Health Sciences, Medical College of Rzeszow University, 35-959 Rzeszow, Poland; 2 Institute of Medicine, Medical College of Rzeszow University, 35-959 Rzeszow, Poland; 3 Department of Gastroenterology with IBD Unit, Clinical Hospital No. 2, 35-301 Rzeszow, Poland

**Keywords:** Crohn’s disease, genetic factors, inflammation bowel disease, pharmacogenetics, ulcerative colitis

## Abstract

Inflammatory bowel disease (IBD) is a chronic disease with periods of exacerbation and remission of the disease. The etiology of IBD is not fully understood. Many studies point to the presence of genetic, immunological, environmental, and microbiological factors and the interactions between them in the occurrence of IBD. The review looks at genetic factors in the context of both IBD predisposition and pharmacogenetics.

## 1. Introduction

Inflammatory Bowel Disease (IBD) is diagnosed in patients with a genetic predisposition who have been shown to have abnormalities of the immune system, usually in correlation with specific environmental factors [1]. They are characterized by periods of active phase of the disease and remission. The course and severity of the disease can vary in patients depending on the location and extent of the inflammation [2]. The most common IBD are ulcerative colitis (UC) and Crohn’s disease (CD). CD and UC can occur in both men and women of all ages. Western lifestyles are causing an increase in the incidence of IBD year after year. Researchers predict that its prevalence will increase significantly in the next few years [3]. IBD was initially diagnosed in western Europe and North America. The authors of numerous publications point to the development of industrialization in these regions of the world as a reason. After 1950, due to a large increase in population and the development of medical care, industry, and the economy, there was also an increase in the incidence of IBD. Subsequently, stabilization of the incidence of IBD was observed in western countries between 1990 and 2015 [4]. Mak et al. in their review present that there has been a sharp increase in the incidence of IBD in the East over the past decade. They also noted that environmental factors and genetic susceptibility differ between eastern and western populations [5]. However, in both western and eastern countries, the cost of IBD in health care systems continues to increase, indicating the need to anticipate future burdens. Due to the aging population and the presence of comorbidities, the period of exacerbation in CD or UC may be longer and more intense [3,6]. The incidence of UC is higher year after year compared to CD. The most common diagnosis of UC is made in the third and fourth decades of life, while CD is diagnosed primarily in people in the second and third decades of life. Up to 16% of IBD cases are diagnosed after age 65, while approximately 4% are diagnosed before age 5. They are most often diagnosed in the Caucasian race. Ashkenazi Jews of Central and Eastern European descent are a particularly vulnerable group, with a four-time higher incidence of CD than others in the Caucasian population. The incidence of IBD varies depending on the geographical location and industrial development of the country. They occur primarily in Europe, especially in Scandinavia and the UK, North America, China, South Korea, Japan, India, and Australia [7].

The etiology of IBD is not fully understood. Several studies point to the presence of genetic, immunological, environmental and microbiological factors and the interplay between them in the occurrence of IBD [8].

## 2. Etiology—Genetics Factors

The first genetic factor that, in 2001, was associated with the occurrence of CD was a mutation in the *nucleotide oligomerization domain containing the protein 2 gene (NOD2).* The *NOD2* gene encodes a protein that functions as a receptor that recognizes components of the building wall of pathogenic bacteria. The pattern recognition receptor (PRR) is found in intestinal epithelial cells and monocytes, where it stimulates their autophagy [9]. The main variants of *NOD2* mutations associated with CD are the following: *R702W* and *G908R* [10]. Relatives with a positive family history for these conditions may show susceptibility to the disease, but this depends primarily on the phenotype of the disease. Other alterations of genes responsible for autophagy, e.g., *ATG16L1—autophagy-related 16-like 1, LRRK2—repeat kinase rich in leucine, 2,* and *IRGM—immune-related GTPase M*, which can predispose to IBD, are also presented in the literature [11,12,13]. IL-10 receptor mutations *(IL10RA* and *IL10RB)* are also examples that can lead to colitis [14,15]. Additionally, some 240 gene loci associated with the predisposition and occurrence of IBD have been found. A direct, shared association with CD and UC has been demonstrated at 30 loci [16]. In addition, some of the loci may be predictive of IBD. In the case of CD, the detected loci include: *FOXO3, IGFBP1,* and *XACT* [17].

### 2.1. Genetic Factors and the Microbiome in Inflammatory Bowel Disease

In recent years, the topic of the influence of genetic factors and the microbiome on IBD has received a lot of attention from researchers [18]. One of the most important challenges in IBD research is to determine how the relationship between genetic factors, immune factors, and the intestinal microbiota, influenced by certain environmental factors, leads to chronic intestinal inflammation. However, intestinal inflammation may be a major factor that strongly influences the composition of the microbiota and therefore it is not obvious to determine the influence of genetics on the intestinal microbiome in people diagnosed with IBD. To date, it is not entirely clear whether dysbiosis (an imbalance or altered composition and function of the microbiota, leading to disrupted host-microbiota interactions) is a cause or a consequence of inflammatory bowel disease [19]. In patients with IBD, there is a decrease in both the diversity of the microbiota species. According to the study, compared to healthy individuals, IBD patients tend to have a greater abundance of members of the *Bacteroidetes* group and *Proteobacteria,* such as *Enterobacteriaceae* (including *Escherichia coli*) and a smaller number of *Firmicutes* (e.g., *Lactobacillus*) [20,21]. A decrease in the number of *Roseburia bacteria* (*Firmicutes cluster*) has been reported in CD patients [22,23]. Patients with IBD patients show a decrease in the number of species that produce butyrate, a short-chain fatty acid that positively modulates intestinal balance and reduces inflammation [24]. It has been documented that the prevalence of specific bacteria, such as *Actinobacteria, Campylobacter species* and *Enterobacteria,* may be important in the development of IBD [25]. Many of the genetic mutations associated with IBD are related to immune function, and in particular with interactions between the immune system and the microbiome. The microbiota affects the activation of certain genes associated with hypomethylated active regulatory regions, thus causing the expression of genes associated with IBD [26]. The susceptibility genes for IBD involved in pathogen recognition and elimination likely influence dysbiosis. These mainly include *NOD2 (nucleotide binding oligomerization domain containing 2), ATG16L1 (autophagy-related 16-like 1), CARD9 (caspase recruitment domain family member 9) and CLEC7A (C-Type Lectin Domain Containing 7A)* [27,28,29,30].

#### 2.1.1. NOD2 (Nucleotide Binding Oligomerization Domain Containing 2)

The strongest genetic risk locus in IBD is the NOD2 gene mutation [31,32]. The protein encoded by the *NOD2* gene is expressed in intestinal epithelial cells (including Paneth cells) and lymphocytes of the lamina propria (including T cells), and most strongly in monocytes and macrophages. It acts as a defense factor against bacteria and contributes to the immune response against commensal microorganisms [33]. It reacts specifically to a peptidoglycan component found in the cell wall of Gram-positive bacteria and Gram-negative bacteria, muramyl dipeptide (MDP), and acts as a potent regulator of cell differentiation, proliferation, and apoptosis through the mitogen-activated protein kinase (*MAPK*) pathway [34]. As a result, mutations in the *NOD2* gene alter Paneth cells’ ability to recognize and eliminate invading pathogens that cause the development of inflammatory bowel lesions [30,34]. Additionally, *NOD2* is required for the initiation of bacterial autophagy, which proceeds with the recruitment of the autophagy molecule *ATG16L1* to the membrane-bound bacterial uptake site. *NOD2* mutations have also been associated with an increase in the number of *E.coli (Proteobacteria type)* and a decrease in the number of *Faecalibacterium prausnitzii (Firmicutes type)* [35]. The *NOD2* gene mutation and its impact on the microbiota in IBD is shown in Table 1.

*NOD2* plays a key role in gut microbiota homeostasis by detecting both commensal and pathogenic microorganisms [45]. In a review by Turpin et al. researchers highlighted the role of *NOD2* in the pathogenesis of IBD by affecting the intestinal microbiota, although the exact mechanism of this phenomenon has not yet been determined [46].

#### 2.1.2. ATG16L1 (Autophagy-Related 16-like 1)

Most of the evidence for an association between this genetic variant and the etiology of IBD comes from functional studies using the variant *ATG16L1 T300A*. The *ATG16L1 T300A* polymorphism is a genetic factor that increases the risk of Crohn’s disease pathogenesis [47]. *ATG16L1* contributes to the regulation of the autophagy pathway, which includes lysosome degradation and intracellular bacterial clearance [48].

*ATG16L1* and *NOD2* interact in the autophagy-dependent antimicrobial pathway, suggesting that defects in both pathways may affect the abundance of *Bacteroides* [49]. Evidence pointing to a role for autophagy in the etiology of IBD comes from genome-wide association studies, which have identified single nucleotide polymorphisms (SNPs) in genes related to autophagy as susceptibility factors for CD. Autophagy is an evolutionarily conserved cellular process in eukaryotes in which cytoplasmic materials are degraded inside the lysosome [50]. This allows the cell to recycle its damaged components and use degraded intracellular materials and proteins in energy production and de novo synthesis [51]. The interaction between genes and their products is essential for the proper elimination of invading pathogens [52]. Loss of *ATG16L1* function alters autophagy in intestinal epithelial cells (Paneth cells) and influences their possibility to secrete various antimicrobial peptides. This promotes bacterial proliferation and invasion through the intestinal epithelium. The *ATG16L1* gene mutation and its impact on the microbiota in IBD is shown in Table 2.

The findings suggest that CD-associated *ATG16L1* polymorphisms *(ATG16L1-T300A, ATG16L1-300)* can alter the composition of the microbiota through changes in antimicrobial peptide secretion, but in a study of 313 patients with IBD, IBD-GRS (based on 200 SNPs associated with IBD risk) showed no association with the composition of the microbiota. These findings suggest that host genetics may influence the composition of the microbiome, but inflammation can mask or alter this relationship [57]. Current data confirm that the *s* polymorphism predisposes to CD, also identifying an additive effect of the G allele in patients with CD, but no association is shown between the ECM1 polymorphisms *(G290S, T130M)* and susceptibility to UC [58]. The results point to a strong genetic background that plays a key role in the development of CD. *ATG16L1 T300A* contributes to intestinal dysbiosis and dysregulated immune responses before the onset of disease symptoms, and autophagy is crucial to maintaining intestinal homeostasis, adequate intestinal immune responses, and antimicrobial protection [59]. The findings show that defective autophagy alone does not cause intestinal dysbiosis, but can do so in combination with an infectious agent.

#### 2.1.3. CARD9 (Caspase Recruitment Domain 9)

*CARD9* also plays a role in the response to bacteria by interacting with *NOD2.* It is selectively expressed in myeloid immune cells, including dendritic cells, macrophages, neutrophils, and consists of an N-terminal activating domain and recruitment caspase *(CARD),* a coiled coil domain, and a C-terminal tail without specific domain structure, and is an important regulator of immunity against bacteria, fungi, and viruses [60]. In 2018, the variant *rs10781499* of *CARD9* was confirmed to be a genetic high-risk factor for IBD, altering the composition of the intestinal microbiota in patients with IBD. *CARD9* deficiency is associated with intestinal fungal dysbiosis, revealing CARD9 signaling as a critical link between intestinal mucosal immunity and intestinal fungi [61]. Some variants of *CARD9* show an increased risk *(rs10870077, rs10781499, and rs4077515),* while others *(rs141992399, rs200735402)* show a protective effect against IBD [62]. This could be attributed to the fact that the *CARD9* variants have different mechanisms of pathogenesis and therefore different susceptibilities to disease. In a study of the Chinese Han population, the *CARD9* predisposing variants *rs10870077* and *rs10781499* do not increase susceptibility to IBD. These divergences can be partly explained by the different prevalence, phenotypes, and epidemiology among patients of the Chinese Han population and western countries [63]. The *CARD9* gene mutation and its impact on the microbiota in IBD is presented in Table 3.

Studies in mice have shown that defects in innate immunity genes, such as *CARD9,* affected the composition of the microbiota. It is not clear how *CARD9* can restore intestinal epithelial homeostasis and restore beneficial bacterial colonization after inflammation. Noting the close relationship between protective genetic variants and therapeutic advances, this may be a good strategy for the rational design of IBD therapy. [19].

#### 2.1.4. CLEC7A (C-Type Lectin Domain Containing 7A)

This gene encodes a member of the *C-type lectin/C-type lectin-like* domain (*CTL/CTLD*) superfamily. The encoded glycoprotein is a small type II membrane receptor with an undulating extracellular *C-type lectin-like domain* and a cytoplasmic domain with a tyrosine-based immunoreceptor activation motif. It acts as a pattern recognition receptor that recognizes various glucans with β-1,3 and β-1,6 bonds from fungi and plants and therefore plays a role in the innate immune response [65]. A recent study showed that bacterial-fungal interactions are crucial in the development of intestinal inflammation [66]. Fungal colonization of the gut is influenced by the bacterial population of *Enterobacteriaceae,* which collaborates with yeast to promote their colonization and active role in intestinal inflammation. These findings also suggest potential therapeutic applications, such as enhancing the protective effects of a probiotic yeast strain, such as *Saccharomyces boulardii CNCM I-745*, or combating *Candida albicans* infection. For example, specific targeting of *Enterobacteriaceae* along with antifungal drugs may be a promising strategy in patients with *Candida albicans* overgrowth. Among the receptors that detect fungal ligands, *C-type lectin receptors (Dectin-1, Dectin-2, Dectin-3, and Mincle)* are the most studied, as they were initially associated with the immune response to fungal infections [67].

A recent review highlighted that most of the properties of *Blautia bacteria* are associated with potential probiotic functions, and the causal relationship between the abundance of *Blautia* and inflammatory bowel disease is not fully understood [68]. In people with CD, *Blautia* counts were significantly reduced in the microbiota of the cecal mucosa compared to healthy subjects [69].

### 2.2. Inheritance of the Microbiota

For many years, twin studies have provided a basis for considering the importance of linking host genes and microbiota inheritance as interdependent hereditary forces that could explain a person’s susceptibility to IBD and even help determine the actual cause of the disease. A study by Turpin et al. involving 271 related healthy individuals from 123 pedigrees found that almost a third of the bacterial taxa in the stool were heritable [46]. Recent publications have identified that the taxon with the highest heritability was *Christensenellaceae* (a family within *Firmicutes*), while *Bacteroidetes* are generally not heritable [61]. In relation to the case of the *Bifidobacterium-lactase* gene locus (LCT), the host’s genetics are likely to shape the microbiome through dietary preferences, which are themselves heritable [70,71]. The finding that there are familial impacts on microbiota constitution implies that specific genetic variants may explain individual variation in microbiota profiles, but this deserves further research. Dysbiosis of the gut microbiota is a characteristic feature of IBD, and both the risk of IBD and the composition of the microbiota are related to genetic and immunological determinants.

### 2.3. Genetic Factors and the Immune System in IBD

The gut is the cluster of the largest number of immune cells in the body, and the gut microbiota directly influences immune function [65]. Various immune mechanisms, such as mucus secretion, immunoglobulin A (IgA) and antimicrobial peptides, shape the intestinal microbiota and prevent direct contact with the epithelium [72]. Subsequent adverse microbial conditions caused by environmental factors can further disrupt immune homeostasis in the intestine, ultimately leading to IBD. The pathways of several key genetic risk factors for IBD (particularly *NOD2* and autophagy) disrupt Paneth cell function, leading to colitis [51,52]. The pathways highlighted by these genes associated with IBD mainly include: microbial detection, activation, and immune suppression. On the basis of current reports, some of the genes are discussed in the following [73].

#### 2.3.1. NOD2

*NOD2* encodes a pattern recognition receptor that is crucial in the immune response [39,41,42]. When the ligand binds, it induces activation of *NF-κB* and *MAPK*, and thus the transcription of pro-inflammatory molecules such as *IL6, IL8, IL1β, TNF-α* [73]. The result is the activation of innate immune cells and the differentiation of acquired immune cells [39]. As mentioned above, *NOD2* is expressed in Paneth cells, suggesting that its polymorphisms may result in defective antimicrobial defense (e.g., reduced release of α-defensin) [74].

#### 2.3.2. ATG16L1 T300A

Autophagy plays a key role in the regulation of the interaction between the intestinal microbiota and innate and acquired immunity, and in host defense against intestinal pathogens [75]. In a study by Lavoie et al., it was shown that the CD *ATG16L1 T300A* risk allele contributes to dysbiosis in mice, specifically due to increased Bacteroides and is associated with increased Th1 and Th17 cells in the lamina propria of the colon and ileum without the development of intestinal inflammation [28]. In a study by Zhang et al. *ATG16L1-deficient* mice showed exacerbated DSS-induced colitis with an increased ratio of pro-inflammatory to anti-inflammatory macrophages, increased production of pro-inflammatory cytokines, and increased numbers of intestinal bacteria coated with IgA [76]. The *ATG16L1 T300A* mutation, by affecting Paneth cells and altering the secretion of antimicrobial peptides, promotes the proliferation of bacteria and their penetration through the intestinal epithelium.

Other autophagy-associated genetic variants, such as *LRRK2* and *IRGM,* have been associated with an increased risk of IBD [11]. They encode repeat kinase 2 proteins rich in leucine and guanosine triphosphate M associated with immunity [77,78,79]. Patients with the *LRRK2* mutation and CD showed increased intestinal dendritic cell activation and thus increased expression and release of pro-inflammatory molecules such as *IL2* and *TNF-α,* while inhibition of *LRRK2* resulted in a reduction of *IL2* and *TNF-α* in patients with CD [70]. *PTPN2 (Protein tyrosine phosphatase non-receptor type 2)* represents an additional gene/protein associated with autophagy initiated by Paneth and lymphoid cells. This results in inappropriate autophagosome formation and impaired bacterial elimination after mutation [80]. High levels of *INF-γ, IL17* and *IL22* have been detected in the serum and intestinal mucosa of patients with *PTPN2* variants [81]. Interestingly, in a recent study by Hoffmann et al., the risk allele of *PTPN2 rs7234029* was clearly associated with a lack of response to anti-interleukin-12/23 treatment (89.9% vs. 67.6%, *p* = 0.005) [82]. In a study by Liu et al. analysis of intestinal bacteria from *ATG16L1 T300A/T300A* mice showed that they had an altered microbiota in both the terminal ileum and the colon compared to wild-type cultured mice. Furthermore, increased production of inflammatory cytokines by immune cells in mice deficient in *ATG16L1* in response to bacteria may activate an adaptive immune response to the intestinal microbiota. *Akkermansia*, a mucin-associated bacterium, was significantly reduced in *ATG16L1 T300A/T300A mice,* and cup cells had reduced mucin secretion resulting from defective autophagy [63].

#### 2.3.3. CARD9

Patients with *CARD9* mutations have primary immunodeficiency disorder (PID), resulting in susceptibility to fungal infections [83]. Several single nucleotide polymorphisms (SNPs) in the human *CARD9* gene are associated with inflammatory diseases, particularly IBD *(rs10781499, rs10870077, and rs4077515). CARD9* controls the virulence of pathogens in a microbiota-independent manner, promoting a specific humoral response [69]. A previously described study by Lamas et al. showed that *CARD9-/-* mice generally exhibit reduced production of inflammatory cytokines, which contributes to the inability to control fungal growth. *IL-6, TNF-α and IL-1β* are cytokines that depend on the function of *CARD9* and protect against fungal infections [64]. Furthermore, *CARD9* mutation in IBD results in a decrease in the production of pro-inflammatory cytokines *(IL-6, TNFα* and *IFNγ*) and Th17 and innate lymphoid cell-ILC-related cytokines (*IL-17A, IL-17F, IL-22)* [84,85,86]. The crucial role of *CARD9* in intestinal immune homeostasis is also highlighted by the impact of *CARD9* signaling on the development of colorectal cancer.

#### 2.3.4. IL23R (Interleukin 23 Receptor)

The *interleukin-23* receptor is a type I cytokine receptor and is encoded in humans by the *IL23R* gene. *IL23R* is expressed in many cell types, including myeloid cells [87]. It is particularly important in maintaining T-cell-dependent immunity and its high levels have been demonstrated in both CD and UC patients. *IL23* is particularly important for the maintenance and development of the Th17 lineage through a positive feedback loop that regulates *IL17, RORγt, TNF, IL1*, and *IL6.* This phenomenon is involved in the expansion of pathogenic pro-inflammatory Th17 cells in CD. Polymorphisms in the gene encoding *IL23R* have been analyzed and linked to the pathogenesis of IBD, indicating an important role for the *IL23/IL17* axis in mucosal inflammation [88]. Furthermore, *IL23* interacts with dendritic cells and macrophages leading to the continued production of various pro-inflammatory molecules, including *IL6, IL12, Il17, INF-γ, TNF-α* and *IL23* itself [89]. The variant *IL23R rs11209026 (Arg381Gln)* has been described as a variant encoding IL23R that protects against IBD, leading to a significantly reduced risk of CD [90]. The presence of alleles *IL-23R rs1004819 and rs11209032* indicates an increased risk of UC in Caucasians [91].

#### 2.3.5. Interleukin 10 (IL-10)

*Interleukin 10* interacts with type II cytokine receptors and is a cytokine with anti-inflammatory effects. It has been shown to modulate both innate and acquired immunity [92]. Mutations in *IL-10* and its *IL10R* receptor have been identified as susceptibility variants to IBD [93]. IL10 inhibits the production of pro-inflammatory cytokines such as interferon-γ, *IL-2, IL-3*, and *TNF-α.* The loss of *IL-10* promotes the development of IBD due to an excessive immune response to the microbiota [94]. A factor associated with *IL-10* is the signal transducer and activator of the transcription 3 *(STAT3)* polymorphism (in loci *rs744166*). This is a factor responsible for the regulation of gene transcription that regulates angiogenesis and cell proliferation. In active CD, an increase in *STAT3* activation is observed in intestinal epithelial cells [95,96]. *IL-10* polymorphisms may be associated with the early onset of colitis [97]. Changes in the *IL-10* sequence have been shown to increase susceptibility to IBD [98,99].

#### 2.3.6. TNFSF15/TL1A (Tumor Necrosis Factor Superfamily Member 15)

*TNFSF15* (encoding a cytokine also known as *Tumor necrosis factor (TNF)-like cytokine 1A (TL1A))* is associated with CD and UC in populations of different ethnicities [100]. The protein encoded by this gene is a cytokine that belongs to the tumor necrosis factor *(TNF)* ligand family and it is expressed in endothelial cells. Expression of this protein is inducible by *TNF* and *IL-1 α.* The expression of *TL1A* expression is related to inflammation levels in IBD. A meta-analysis by Zhang et al. found significant associations between six *TNFSF15* polymorphisms and CD risk *(rs3810936, rs6478108, rs4979462, rs6478109, rs7848647, rs7869487)* with the exception of the *rs4263839* polymorphism. Polymorphisms *rs3810936, rs6478108,* and *rs6478109* were significantly associated with the risk of UC. Ethnic differences had no effect on risk [101]. There is now evidence that interactions between *TL1A* and its functional receptor *DR3 (death domain receptor 3)* affect intestinal mucosal immunity under both homeostatic conditions and various inflammatory conditions [102]. In a review by Valatas et al. the authors highlighted that the interpretation of the role of the *TNFSF15* mutation is influenced by ethnicity, as a stronger association with Asians was reported [103]. The study by Liu et al., which included patients with IBD and healthy individuals in Europe and Asia, found that although allele frequencies in European and Asian populations are similar, *TNFSF15* variants have a stronger association with IBD susceptibility in Asian individuals [104]. Other researchers have suggested that certain *TNFSF15* alleles may have predictive value for the severity of IBD. In the Chinese population, the polymorphism *rs10114470* allele was associated with an increased probability of constrictive, penetrating, or perianal complications [105]. *DR3* signaling enhances CD4+ lymphocyte proliferation by increasing both IL-2 production and *IL-2RA* and *IL-2RB* expression [106]. *TL1A* preferentially increases mouse memory CD4+ T cell proliferation, but can also induce mild proliferation and strong expression of *IL-2* and *IFN-γ* by naive T cells. *TL1A* costimulation of *CD4+ T* lymphocytes results in the production of multiple cytokines, including *IL-2, IL-4, IL-13, interferon-γ (IFNγ)* and *IL-17* [107,108,109]. *TL1A* enhances many immune pathways that, if maintained, may be involved in the development of fibrosis. *Interleukin 17A* promotes fibrosis in experimental models of pulmonary and cutaneous fibrosis and has been found to be overexpressed in intestinal stenosis in patients with CD [107]. *IL-13* is associated with experimental mouse intestinal fibrosis, acting mainly through *TGFβ,* and is also increased in constriction in patients with CD. elevated expression of *TL1A* and *DR3* was found in the *SAMP1/YitFc* mouse model of ileitis, which is phenotypically associated with the development of overt intestinal strictures [108]. In human fibrotic conditions, including IBD, the immune and profibrotic events responsible for *TL1A* are not sufficiently studied. The potential impact of *TL1A* on these pathways was highlighted in a report on *TL1A* expression by human intestinal myofibroblasts isolated from patients with IBD. *TL1A* expression by intestinal myofibroblasts was upregulated by pro-inflammatory cytokines *(IFN-γ, TNF-α, IL-1α)* or intestinal tissue culture supernatants from IBD patients [109]. A summary of the described genes versus immune system in IBD is presented in Table 4.

Immune dysregulation, microbial dysbiosis, and environmental factors are considered key factors in IBD, but the family history of the disease also plays a key role.

### 2.4. Practical Implementation of Genetics in IBD

One of the strongest risk factors for the development of IBD is a positive family history, which may have some influence on the phenotype of IBD [110,111]. According to Santos et al., it occurs in approximately 8-12% of patients with IBD [112]. Other studies indicate that this level is averaged between 4.5-14.5%, and according to Chao et al. it affects up to 20% of patients with IBD [113,114,115,116]. The greatest finding in this regard was a 34-year cohort study in the Danish population, in which Moller et al. showed that the highest incidence rate of IBD occurred in first-degree relatives (FDR) (almost 8 times higher for CD and 4 times for UC), second- and third-degree relatives, and the highest risk was observed early in life (especially <20 years of age) [105]. Current researchers confirm that IBD with a positive family history is associated with a younger age of diagnosis and a more unfavorable disease phenotype [117]. This is supported by research findings and indicates that the prevalence of a positive family history of IBD in the pediatric population is increasing [118,119]. Children diagnosed between 2016 and 2020 had a higher prevalence of positive family history compared to those diagnosed between 2010 and 2015 (31.8% vs. 20.7%, respectively, *p* = 0.024) [120]. However, some articles show different data, suggesting that there is no association between the onset of IBD and multigenerational consanguinity [120]. Families with multiple affected individuals are more likely to have a compatible type of disease (CD or UC), but 25% of cases find patients with two different types of disease in the same family [121]. Some studies have shown that a positive family history of CD is an independent risk factor for intestinal resection and the use of tumor necrosis factor (TNF) therapy, but some reports are contradictory and most studies have not determined the concordance of the type of IBD and the strength of the family history [122,123].

#### 2.4.1. Different Degrees of Relatedness in IBD

Studies on the risk of familial IBD indicate an increased risk of CD and UC in first-degree relatives. According to Kevans et al., a higher genetic risk of IBD is observed in CD in first-degree relatives and the genetic profile of affected CDs is enriched with IBD risk alleles (*rs2188962* in the *IBD5 locus* region and *rs3764147* in the *LACC1* region) compared to controls [124]. In a study by Hoffmann et al. 16.6% of the patients indicated having at least one first degree relative who also suffered from IBD [82]. According to Gabbani, about 5%-23% of patients have at least one first degree relative with IBD [125]. In a prospective study by Capone et al., the prevalence of a positive family history increased from 13.7% at diagnosis to 26.6% after 20 years in first-degree relatives and from 38.5% to 52.2% in all relatives. At 20 years of follow-up, an additional 10% of the probands had a sibling, 6.1% had a parent, 1.9% had a grandparent and 4.5% had a cousin diagnosed with IBD [126]. Current results also indicate that offspring have an earlier onset and more severe form of the disease compared to parents. In a study conducted in central China, the median age of diagnosis was lower in patients with a positive family history (29.0 vs. 36.0 for CD; 35.5 vs. 41.0 for UC) [127]. Ballester et al. obtained similar results, also showing that the median age at diagnosis was lower in the group with a positive family history. Ballester et al. obtained similar results, also showing that the median age at diagnosis was lower in the group with a positive family history (32 vs. 29, *p* = 0.07), and 14.2% of the patients had relatives with IBD [128]. In the observation by Halfvarson et al., those with IBD compared to controls were more likely to have a mother, father, full siblings (and a child with IBD [129]. The strength of the association increased with the number of first-degree affected relatives and was modified by the IBD subtype and the age of diagnosis. In a study by Borren et al., a family history of IBD was found in 32% of the patients studied (*n* = 2094), 17% had an affected first-degree relative, and 21% had a second-degree relative [130]. However, there was an earlier age of diagnosis only if the affected family member had UC [= but not CD. In the univariate analysis, CD patients with a positive family history were more likely to have complicated disease [56%, especially the association was observed in siblings. In particular, studies of twins and families for IBD have shown that a child has a 26-fold higher risk of developing CD when another child already has it, and the risk is 9 times higher for UC [131]. A study by Park et al. also confirms a higher concordance of the type and phenotype of IBD in first-degree relatives than in second- or third-degree relatives [132]. Furthermore, the risk of IBD in the offspring increases significantly if both parents have IBD, with an estimated risk, depending on observation, of 33-52% [133]. This relationship was challenged by Mouzan et al. in a study of 138 children, which did not show an association between parental consanguinity and IBD in the children studied [134]. A study comparing the prevalence and family history of IBD between China and the United States showed that the three most common types of relatives affected by IBD were cousin, sibling, and parent in the United States compared to child and sibling in China [135]. Interestingly, dysbiotic changes have been observed in healthy first-degree relatives of IBD patients with an associated increase in fecal calprotectin (a biomarker of intestinal inflammation) [136,137]. The location (loci) of CD susceptibility genes within chromosomes 1, 3, 4, 5, 6, 7, 10, 12, 14, 16, 19 and X is also known [138,139,140,141,142,143,144]. The regions on chromosomes 16, 12, 6, 14, 5, 19 have been confirmed and named *IBD1* through *IBD7*, respectively [145]. Of these seven loci, only *IBD1* (chromosome 16q12) has been replicated in all studies, while *IBD2* (chromosome 12), *IBD3* (chromosome 6) and *IBD4* (chromosome 14) have been replicated in some studies [146].

Some genes are involved in the development of both CD and UC *(3p21.31, NKX2-3, CCNY).* In addition, other genes, ie *HERC2, ECM1, STAT3,* and *PTPN2,* have been linked only to UC but not to CD. Furthermore, to date, more than 20 exclusive UC loci have been recognized, including *ARPC2, IL10* and *ECM1,* among others [147,148,149,150].

#### 2.4.2. Inflammatory Bowel Disease in Twins

Twin cohorts are often used to study the heritability of diseases. In IBD, twins are the group with the highest risk of developing the disease; in addition, twin studies also allow control of genetic variation, microbiome, and epigenome. Studies of twins have provided the best evidence for a genetic predisposition to IBD, which is stronger in CD than in UC. The main premise of twin studies is that pairs of twins share a similar environment and consistent genetic variation, with monozygotic twins (MZ) sharing 100% of segregating genes and dizygotic twins (DZ) sharing 50% [151]. Studies have shown that concordance rates are significantly higher in monozygotic twins than in dizygotic twins for both CD and UC [105]. Similarly, in a study by Annese et al., the concordance in the diagnosis of UC (15.3 vs. 3%) and CD is higher in monozygotic twins than in dizygotic twins [17]. According to Bell et al. MZ twins share common genotypes and epigenetic profiles at conception [121]. Furthermore, it should be considered that some epigenetic differences emerge during the lifetime of MZ twins, but interindividual differences with respect to the epigenome and intestinal microbiota remain smaller between twin pairs than between unrelated individuals [152]. An evaluation of cohorts of twins from various countries, for example Norway, suggests that environmental factors are more important than previously thought and that the lifetime concordance rates of pairs of twins with MZ never reach close to 100% [153]. A current analysis of the UK IBD Twins Registry showed significantly higher concordance for CD between pairs of MZ twins compared to pairs of DZ twins. In addition, the data also showed a trend towards a higher concordance of MZ pairs in UC, although statistical significance was not reached [154]. This is consistent with other cohorts of twins and indicates the heritability of CD. A recent report showed that the gut microbiome of healthy twins from pairs of discordant twins with IBD showed signatures similar to IBD, indicating that discordant monozygotic twins with IBD share not only the same genetic background, but also the same gut microbiome [155]. Monozygotic twins with UC incompatibility have also been shown to differ in the bacterial composition of their gut microbiota, and affected twins show less diversity than their healthy twins [156]. Interestingly, a study of monozygotic twins of different ages found that the immune systems of MZ twins became increasingly divergent at later ages, suggesting that the immunological differences are mainly due to environmental and non-hereditary factors [157]. New genes have also been identified that could potentially play a role in the pathogenesis of IBD among twins. *CYP2C18* expression has been observed to be strongly up-regulated in IBD affected twins [158]. Studies on the familial occurrence of IBD provide valuable information not only for researchers but also for healthy relatives who are naturally concerned about the risk of the disease. In addition to genetic factors, environmental factors, intestinal microbiota composition and role of the immune system; epigenetics is also involved in the pathophysiology of IBD.

### 2.5. Epigenetics

Epigenetics deals with the study of mechanisms that affect hereditary changes in phenotype, regardless of the DNA sequence [54]. It probably plays a role in the pathogenesis of IBD, as certain environmental factors can influence the risk of developing IBD through epigenetic modifications, such as DNA methylation, post-translational modifications of histones, and the expression of noncoding RNAs [159]. Epigenetic mechanisms have been shown to play an important role in several fundamental biological processes, including cell differentiation, transcription of cell type-specific genes and cell function, and immune memory [160].

#### 2.5.1. DNA Methylation

DNA methylation is the most common and best studied epigenetic direction of IBD [161,162,163]. Moret-Tatay et al. in 2019 identified an epigenetic methylation signature (methylation status of the *DEFA5* and TNF genes as a characteristic biomarker) to characterize CD patients and support the likely influence of the environment and immune system on CD pathogenesis [164]. Considering that some environmental factors may mediate epigenetic regulation, many researchers have described the diet as one of the risk factors for IBD. An example described by Rapozo et al. is single-carbon metabolism dependent on dietary components (e.g., methionine, betaine, and folic acid), which are involved in DNA methylation pathways and methyl group supply [165]. In addition, Silva et al. showed that butyrate deficiency may be partly responsible for excessive condensation of chromatin structure and histone deacetylase-mediated gene expression [166]. Interestingly, melatonin and a probiotic *(Bifidobacterium longum),* may regulate the DNA methylation status of intestinal epithelial cells [167,168]. Pan et al. demonstrated the influence of the microbiota on the maturation of DNA methylation features and changes in the transcriptome. They found that it can regulate the intestinal transcriptome during postnatal development and, through its corresponding DNA methylation state, targets a portion of genes that respond to the microbiota [169]. Ansari et al. showed that the microbiota affects the activation of some genes associated with hypomethylated active regulatory regions, thus inducing the expression of genes associated with colitis and IBD [170]. Hypomethylation of ribosomal protein kinase S6 A2 *(RPS6KA2*) has also been identified as a diagnostic aid in predicting the complex disease behavior (constrictive/penetrating disease) of CD and extensive UC disease [171]. *RPS6KA2* is responsible for modulating cell growth, motility, and proliferation and regulating the *PI3K/Akt/mTor* pathway and autophagy. Autophagy has been shown to be one of the most important pathogenesis of CD.

An analysis by Kraiczy et al. demonstrated the role of DNA methylation in the regulation of human intestinal epithelial development and function. They confirmed the developmental origin of IBD through epigenetic mechanisms by overlapping genomic loci that undergo significant changes in DNA methylation during gastrointestinal development with loci observed in children diagnosed with IBD [172]. Howell et al. performed a complete genome analysis that revealed differences in intestinal segment and disease-specific differences, DNA methylation, and transcriptional changes in human epithelial cells obtained from mucosal biopsies from pediatric patients with IBD, compared to healthy controls [161]. A study involving 18 CD patients compared to 25 healthy controls identified 4287 differentially methylated positions in DNA derived from peripheral blood cells, indicating that CD patients exhibit, as the authors put it, “a specific methylation landscape” [173]. A study by McDermott et al. that evaluated genome-wide DNA methylation changes associated with CD and UC and IBD activity showed that in the case of CD, the most identified pathways include immune responses, regulation of T cell activation, and cellular responses to bacterial-derived molecules, suggesting that epigenetic modification is involved in the dysregulation of bacterial and immune responses in IBD [174]. A recent study by Venkateswaran et al. among pediatric patients, mostly of European origin, focused on identifying quantitative trait loci (mQTLs) or SNPs that bind to DNAm at specific CpG sites. Blood mQTLs have been shown to be reproducible, supporting their generalizability across age groups, ancestry, disease status and DNA sample source, and provide a valuable resource for future research on blood mQTLs [175].

Furthermore, IBD increases the risk of colorectal cancer (CRC), which has a genetic basis, but epigenetics may also be a factor in the disease [176]. Rajamäki et al. showed that there was global hypermethylation in IBD-associated CRC (IBD-CRC) compared to sporadic CRC clustered separately. There was no association with younger age at diagnosis or differential expression of methylation-regulating enzymes, suggesting that these methylation changes are due to inflammation [177]. A recent meta-analysis of research studies indicated that methodological homogenization of IBD epigenetic studies is necessary to allow consolidation and independent validation, and an indicated direction for future research was the identification of epigenetic biomarkers of peripheral blood leukocyte DNA methylation in IBD [178].

#### 2.5.2. Histone Modifications

Another epigenetic mechanism of transcriptional regulation involves post-translational modifications of histone proteins (also known as histone modifications). Histone modification plays an important role in the occurrence and development of IBD. Among post-translational histone modifications, acetylation and methylation are the most studied post-translational modifications [179,180]. Histone modification in IBD may be an indicator of microbiota-host interactions. The decrease in *Roseburia* abundance in the gastrointestinal tract of patients with UC is associated with a reduced methylation of the *KHDC3L* gene. These findings suggest that epigenetics and the gut microbiota may work together to regulate the occurrence of IBD [148]. Because *p62* is involved in the inflammatory response and is elevated in patients with IBD, and histone modification plays an important role in regulating *p62* expression, a study by Chen et al. elucidated the epigenetic mechanism by which *SETD8* regulates *p62* expression and suppresses the inflammatory response in colitis. The authors suggest that targeting *SETD8* may be a promising therapy for IBD [181].

#### 2.5.3. Expression of Noncoding RNAs

In addition to the described mechanisms, epigenetic regulation can also involve noncoding ncRNAs, which, depending on their length, can be divided into lncRNAs, mncRNAs, sncRNAs, and microRNAs (miRNAs). MicroRNAs are a group of noncoding RNAs that mediate RNA silencing and gene expression. ncRNAs are differentially expressed between controls and IBD patients, and there is also a difference in expression between CD and UC patients [182]. In IBD, miRNAs are involved in the regulation of the intestinal mucosal barrier, T cell differentiation, the Th17 signaling pathway, and autophagy, so recent studies have focused on microRNAs [183,184,185,186]. MiRNA has been found to be a key regulator of intestinal immunity and is involved in innate immunity and adaptive immunity. In response to inflammation, miRNAs can affect the maturation and differentiation of immune cells. For example, bone marrow-derived miRNA-223 can reduce *IL-1β* release by inhibiting *NLRP3*, thus alleviating colitis in mice [187]. Exosomes containing miRNA-155 are released into the gastrointestinal tract, host macrophages are induced to polarize towards M1, and colitis increases [188]. Short-chain fatty acids-SCFAs can also promote miRNA expression in B cells and regulate B cell differentiation [189,190]. In addition, the host can also regulate the structure and growth of the intestinal flora through miRNAs. Liu et al. showed that miR-30d in feces can attack *Akkermansia muciniphila* and increase the abundance of modified bacteria by up-regulating lactase expression [63]. Furthermore, some miRNAs, such as miR-199a-5p and miR-1226, can play an important role in the interaction between the host and the microbiota, providing new ideas to maintain intestinal homeostasis.

According to the study, miRNA-146a can alleviate colitis by targeting the *TRAF6* and NF-κB signaling pathways and can inhibit the activation of pro-inflammatory macrophages (M1), as well as the production and release of pro-inflammatory factors through the TLR4 pathway [191,192]. Furthermore, the results show that miR-223 expression is positively correlated with the activity of UC disease. Fecal miR-223 can distinguish well between patients with IBD in active and remission phases, with a sensitivity and specificity of 80 and 93%, respectively [193]. A prospective study by Kalla et al. reports that miR-3615 and miR-4792 in blood T cells contribute to the prognosis of UC [194]. However, most current studies focus only on detecting the changing trend in miRNA expression, and further quantitative analyzes are still needed. In the current literature, a developing research avenue is lncRNA, which plays an important role in the pathogenesis of IBD [195,196,197,198]. New reports indicate that interferon γ-antisense 1 (*IFNG-AS1*), which is elevated in the intestinal mucosa of patients with IBD, is a mediator of the inflammatory signaling cascade in the pathophysiology of IBD [160,199].

The epigenetic mechanisms described contribute to the development, progression, and maintenance of IBD. They are typically triggered by a number of environmental factors. Some authors list three critical periods in which the environment can promote the onset of IBD; these are: the prenatal period (in response to the mother’s lifestyle), the early postnatal period (during colonization of the intestinal microbiota), and just before the onset of the disease [200]. Epigenetics is an important and needed research direction in the pathogenesis of IBD, and the identification of epigenetic signatures in IBD may help develop new clinical biomarkers of the disease.

### 2.6. Pharmacogenetics in Inflammatory Bowel Disease

Researchers are increasingly identifying genetic factors as an extremely important component of the appropriate susceptibility of the body to selected treatments for IBD. Through the development of pharmacogenetics, specific types of treatment have begun to be studied with specific types of drugs in patients. One such drug is Infliximab, which was studied by Arijs et al. and they linked the expression of five mucosal genes in UC to the lack of therapeutic response after Infliximab [201]. McGovern et al. present in their article that it is extremely important to know the pathways of therapeutic intervention to establish effective therapy in IBD [202]. The response to anti-TNF-α therapy depends on both TNF-α and TNFR(TNF Receptor) polymorphisms (A allele in -308 TNF-α and G allele in *TNFRSF1A*), as well as equal types of immune and cytokine pathway polymorphisms [203]. Van den Bosch et al. indicate that metabolic reactions between intestinal microbes, as well as lipid and amino acid metabolites, can also affect the efficacy of anti-TNF therapy [204]. However, Zhang et al. in their study noted that the efficacy of glucocorticosteroid (GC) treatment may depend on DNA methylation (DNAm), confirming that the systemic response may depend on epigenetic mechanisms [205]. Furthermore, Lucafo et al. indicate that long noncoding RNA (lncRNA) *GAS5* has been shown to reduce sensitivity to GC treatment [206]. In addition, high expression of glucocorticoid receptor β steroid resistance in patients with UC [207]. Azathiopurine (AZA), 6-mercaptopurine (6-MP), which are among the thiopurine compounds, have been widely studied. Park et al. in their article present, among other things, that the polymorphism of the thiopurine S-methyltransferase (*TPMT*) allele is associated with a poorer therapeutic response of AZA and 6-MP [208]. It is associated with high levels of *6-TGN* (6-thioguanine nucleotide), which can lead to the development of myelotoxicity [209]. In addition to the *TPMT* polymorphism (TPMT *1/*3C), NUDT15 p.Arg139Cys (nucleoside diphosphate-linked moiety X-type motif 15, p.Arg139Cys variant) may be associated with the appearance of leukopenia [210]. Ye et al. in their review article indicate that up to 14% of Europeans who undergo thiopurine treatment with the *TPMT* polymorphism have myelosuppressive episodes [211]. Therefore, it is important to measure the metabolites and enzymes associated with thiopurines to reduce the risk of complications [212]. In another study, the authors focused on anti-integrin therapy used in UC patients. They showed that the expression of the integrin αE gene (*ITGAE*) and granzyme A (*GZMA* mRNA) may be associated with the response to etrolizumab therapy [213]. Personalized medicine, which is based on pharmacogenetics and therapeutic drug monitoring (TDM), should be an integral factor in the treatment of patients with IBD [214]. 

## 3. Conclusions

There is a year-on-year increase in the incidence of IBD, especially in eastern countries. Researchers are increasingly looking at genetic factors linked to the gut microbiota that may predispose to UC or CD. In addition to *NOD2,* strong correlations have been detected between *ATG16L1 T300A, CARD9, CLEC7A,* and IBD. Genetic factors and altered intestinal microbiota are also associated with an intensification of the immune system in patients with IBD, during the exacerbation of the disease. Furthermore, recent scientific reports also point to important epigenetic mechanisms that are involved in selected basic biological processes and probably play a significant role in the pathogenesis of IBD, but more research is needed in this area.

The gene and its polymorphisms associated with inflammatory bowel disease are described in Supplementary Table S1.

## Figures and Tables

**Table 1:** The *NOD2* gene mutation and its impact on the microbiota in IBD.

	Genes Studied	Conclusions of the Study
Animal studies	*NOD2* gene	In mouse models of colitis, the *NOD2* gene mutation is associated with dysbiosis, which does not cause changes in mucosal or immune tissue [36]. Mice with *NOD2* gene mutation show inefficient recognition and clearance of bacterial pathogens. They have been observed to have statistically significant increases in *Porphyromonadaceae (Bacteroidetes), Enterobacteriaceae, Sutterellaceae (Proteobacteria)* and *Coriobacteriaceae (Actinobacteria)* [37].
Research involving humans	*NOD2* gene	In 474 patients with IBD, there was an association between the dose of *NOD2* risk alleles, consisting of *rs104895431, rs104895467, rs2066844, rs2066845, rs5743277, rs5743293*, and the increased relative abundance of *Enterobacteriaceae.* The association may be independent of the disease and may play a role in the pathogenesis only in individuals with other risk factors [38]. Carriers of the C allele at *rs2066845* were significantly associated with an increase in relative abundance in the fecal bacterial family *Erysipelotrichaceae. NOD2* polymorphisms contribute to the composition of the fecal microbiome in asymptomatic individuals. It is unknown whether this modulation of the microbiome affects the future development of CD [39]. An association has been confirmed between the ileal phenotype of CD-affected individuals with a reduced relative abundance of the *Ruminococcaceae* family and an increased relative abundance of the *Actinobacteria* group and the *Firmicutes/Bacillus* class [40]. In a cohort of 1,514 healthy individuals, the *NOD2* locus was associated with the enterobactin biosynthesis pathway, which is highly correlated with the abundance of *E. coli.* The authors suggested that enterobactin produced by *E. coli* inhibits myeloperoxidase (MPO), a bactericidal host enzyme, thus providing a survival advantage that allows *E. coli* to bypass the innate host immune response in inflammatory bowel disease [41]. An increase in anaerobic bacteria from the *Enterobacteriaceae* family has been shown in the microbiota of CD patients compared to controls without IBD [42]. The *ATG16L1 (Autophagy-related 16-like 1 protein)* and *NOD2* genes probably played an essential role in the beneficial immunomodulatory properties of *Bacteroides fragilis,* which protects mice from experimental colitis [43]. *Candida albicans* was shown to be the most abundant species in CD-positive patients, as well as in those with CD-associated *NOD2* mutations. More studies have been required to determine whether *Candida* is an intestinal commensal or a pathogen in patients with CD [44].

**Table 2:** The *ATG16L1* gene mutation and its impact on the microbiota in IBD.

	Genes Studied	Conclusions of the Study
Animal studies	*ATG16L1* gene	There were changes in the composition of the fecal microbiota of knock-in mice expressing the variant *ATG16L1 (T300A*) compared to wild-type (WT) mice. An increase in the number of *Bacteroides (Bacteroides ovatus)* was demonstrated. During dextran sulfate sodium (DSS)-induced colitis, knock-in mice show an altered fecal microbial composition associated with a decrease in *Firmicutes* and an increase in *Bacteroidetes, Proteobacteria,* and *Cyanobacteria* compared to WT mice. Changes occurred before the onset of the disease, suggesting that *ATG16L1 T300A* contributes to dysbiosis before the onset of IBD symptoms [28]. Mice with *ATG16L1 (T300A)* mutations show reduced antimicrobial autophagy and abnormal lysozyme distribution in Paneth cells [53]. *ATG16L1* mice show morphological abnormalities in Paneth and cup cells, but do not develop intestinal autophagy or inflammatory bowel disease [54]. *ATG16L1 T300A/T300A* mice showed a significant increase in the abundance of *Tyzzerella, Mucispirillum, Ruminococcaceae* and *Cyanobacteria* in both feces and intestinal mucosa, while *Akkermansia,* a mucin-associated bacterium, was significantly reduced in *ATG16L1 T300A/T300A* mice. Dysbiosis in *ATG16L1 T300A* mice may be an important factor that contributes to increased susceptibility to IBD [55].
Research involving humans	*ATG16L1* gene	The inflamed ileum of patients homozygous for the *ATG16L1* allele *(ATG16L1-T300A)* contained an increased number of *Fusobacteriaceae,* while the inflamed ileal tissue of patients homozygous for the protective allele of *ATG16L1 (ATG16L1-300)* showed a decreased number of *Bacteroidaceae* and *Enterobacteriaceae* and an increase in *Lachnospiraceae.* [56].

**Table 3:** The *CARD9* gene mutation and its impact on the microbiota in IBD.

	Genes Studied	Conclusions of the Study
Aminal studies	*CARD9* gene	Disturbed microbial tryptophan metabolism in *CARD9* was associated with susceptibility to colitis. Administration of *Lactobacillus* strains that metabolize tryptophan to AHR ligands was sufficient to reduce colitis in *CARD9 -/-* mice. The gut microbiota of CARD9-/- mice has a pro-inflammatory effect because it enhances colitis when transferred to germ-free wild-type mice [61]. *CARD9* effectively controlled staphylococcal virulence to promote pathogen elimination through gut microbiota-independent and -dependent mechanisms. Mice with induced colitis had lower levels of *Clostridiaceae* and higher levels of *Firmicutes* in the basal composition of fecal bacteria. The fecal fungal composition of *CARD9-/-* mice was altered and dominated by representatives of the *Ascomycota, Basidiomycota*, and *Zygomycota* types. Genetic susceptibility to intestinal pathogens can be counteracted by dietary intervention, which restores humoral immunity and a competitive microbiota [64].

**Table 4:** Summary of genes described versus immune system in IBD.

Gene Group	Name of the Gene	Function
Genes associated with molecular pattern recognition of pathogens	*NOD2/CARD15, CARD9*	Activation of pro-inflammatory and anti-inflammatory cytokines and regulation of inflammation and cell apoptosis.
Genes associated with autophagy	*ATG16L1, IRGM, LRRK2*	Key role in regulating the interaction between the gut microbiota and innate and acquired immunity, and in host defense against intestinal pathogens.
Genes associated with lymphocyte differentiation	*IL23R*	Activation and development of the Th17 lineage and its effects on dendritic cells and macrophages leading to the production of various pro-inflammatory molecules.
Genes encoding interleukins	*IL-10*	An anti-inflammatory cytokine that inhibits the production of pro-inflammatory cytokines.
Genes encoding the protein	*TNFSF15*	The TNFSF15 gene product (TL1A) is a TNF-like factor that is expressed in endothelial cells, macrophages and lymphocytes of the intestinal lamina propria.

## Data Availability

Not applicable.

## Supplementary Materials

The following supporting information can be downloaded at: https://www.mdpi.com/article/10.3390/genes13122388/s1, Table S1: Gene and its polymorphysms connected with inflammatory bowel disease.
