# Supplementary material for: Genetic and Epigenetic Etiology of Inflammatory Bowel Disease: An Update
Source: Genes (Basel). 2022 Dec 16;13(12):2388. doi: 10.3390/genes13122388 (PMC9778199; doi:10.3390/genes13122388)
Supplement: Supplementary file 1 [file genes-13-02388-s001.zip › genes-2072038-supplementary.pdf]

Table S1. Gene and its polymorphysms connected with inflammatory bowel disease.

The Gene and Its Polymorphysms Connected with IBD	Variants of Gene	Conclusions of the Study	References
NOD2 (Nucleotide Binding Oligomerization Domain Containing 2)	Polymorphisms at loci: *rs104895431, rs104895467, rs2066844, rs2066845, rs5743277, rs5743293*	Increase in the abundance of *E. coli (type proteobacteria), Porphyromonadaceae (Bacteroidetes), Enterobacteriaceae, Sutterellaceae (type proteobacteria), Coriobacteriaceae (Actinobacteria),* the *Firmicutes/Bacillus* class, and the *Actinobacteria group.*	36-44
ATG16L1 (Autophagy-Related 16-like 1)	*ATG16L1-300, ATG16L1 T300A*	*ATG16L1* mutations show reduced antimicrobial autophagy and abnormal lysozyme distribution in Paneth cells.<br><br>Decrease in the abundance of *Firmicutes* and an increase in increase in *Bacteroidetes, Proteobacteria, and Cyanobacteria.*<br><br>*The ATG16L1 allele* (*ATG16L1-T300A*) contained an increased number of *Fusobacteriaceae*, while the inflamed ileal tissue of patients homozygous for the protective allele of *ATG16L1 (ATG16L1-300)* showed a decrease in the number of *Bacteroidaceae* and *Enterobacteriaceae* and an increase in *Lachnospiraceae.*	36, 53-56
CARD9 (Caspase Recruitment Domain Family Member 9)	Some variants of *CARD9* show an increased risk (*rs10870077, rs10781499, and rs4077515*), while others (*rs141992399, rs200735402*) show a protective effect against IBD.	*CARD9* controls the virulence of pathogens in a microbiota-independent manner, promoting a specific humoral response. Disturbed microbial tryptophan metabolism and dysbiosis. Important regulator of immunity against bacteria, fungi, and viruses.	69,64
CLEC7A (C-Type Lectin Domain Containing 7A)	-	It acts as a pattern recognition receptor that recognizes various glucans with β-1,3 and β-1,6 bonds from fungi and plants and therefore plays a role in the innate immune response.	67
IL23R (Interleukin 23 Receptor) gene	Polymorphisms at loci *rs11209026*	It has been described as a variant encoding *IL23R* that protects against IBD, leading to a significantly reduced risk of CD.<br><br>Indicates an increased risk of UC in Caucasians.	90,91

	Polymorphisms at loci *rs1004819, rs11209032, rs11209032*		
LRRK2 (Leucine Rich Repeat Kinase 2)	-	*The LRRK2* mutation and CD showed increased intestinal dendritic cell activation and therefore increased expression and release of pro-inflammatory molecules such as IL2 and TNF-α, while inhibition of *LRRK2* resulted in a reduction in decreased production of IL2 and TNF-α production in CD patients.	70
STAT3 (Signal Transducer and Transcription Activator 3)	Polymorphism in loci *rs744166*	This is a factor responsible for the regulation of gene transcription transcription of genes that regulate angiogenesis and cell proliferation. In active CD, an increase in *STAT3* activation is observed in intestinal epithelial cells	95,96
TNFSF15/TL1A (Tumor Necrosis Factor Superfamily Member 15)	Polymorphism in loci: *rs3810936, rs6478108, rs4979462, rs6478109, rs7848647, rs7869487, rs3810936, rs6478108, rs6478109*	The protein encoded by this gene is a cytokine that belongs to the TNF family of ligands and is expressed in endothelial cells. The expression of this protein is inducible by TNF and IL-1 α. Expression of *TL1A* is related to inflammation levels in IBD.	101
PTPN2 (Protein Tyrosine Phosphatase Nonreceptor Type 2)	Polymorphism in loci *rs7234029*	High levels of INF-γ, IL17 and IL22 have been detected in the serum and intestinal mucosa of patients with variants of *PTPN2.* The risk allele of *PTPN2* was clearly associated with a lack of response to anti-interleukin-12/23 treatment.	81,82
